# Do Great Apes Use Iconic Gestures?

**DOI:** 10.1002/wcs.70022

**Published:** 2026-02-08

**Authors:** Marcus Perlman

**Affiliations:** ^1^ Department of Linguistics and Communication University of Birmingham Birmingham UK

## Abstract

Many researchers in cognitive science and linguistics now recognize that iconicity—perceived resemblance between the form and meaning of a signal (e.g., a word, sign, or gesture)—is an essential property of language, playing vital roles in its processing, learning, and historical development. Iconicity is also fundamental to the human ability to create meaningful new signals without reliance on convention. This iconic turn raises a critical question for the study of language origins: Do great apes use iconic gestures? Apes are well documented to use a flexible and wide‐ranging repertoire of gestures, and many appear to be iconic representations of actions, including directive touches, visual directives, and pantomimed actions. However, the most widely accepted theories—ontogenetic ritualization and biological inheritance through phylogenetic ritualization—argue that this apparent form‐meaning resemblance is not psychologically real to the apes using the gestures. They argue instead that effective actions are channeled into gestures through repeated use, either through an individual's experience or over generations of evolution. Yet, it is increasingly recognized that these theories cannot account for the variability and contextual tuning of ape gestures. Alternatively, reasoning from cognitive theories of human gesture and iconicity as rooted in sensorimotor simulation and mental imagery, apes may use a range of gestures that appear homologous to the iconic gestures of humans, even if comparatively restricted in imaginative scope and anchored heavily in a here‐and‐now context. This fundamental capacity for iconic gesturing may have been a critical precursor to the evolution of language.

## Graphical abstract

Iconicity is increasingly recognized as a core property of language, raising the evolutionary question of whether great apes use iconic gestures. This article challenges current theories of ape gesturing, arguing that many ape gestures are homologous to human iconic gestures, indicating a crucial role for iconicity in language evolution.

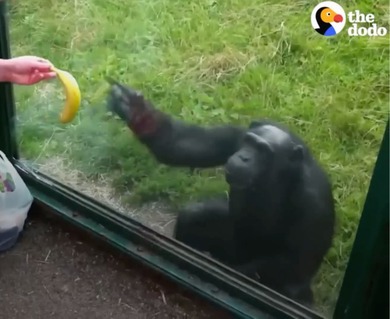

## 1 Introduction

**FIGURE 1:**
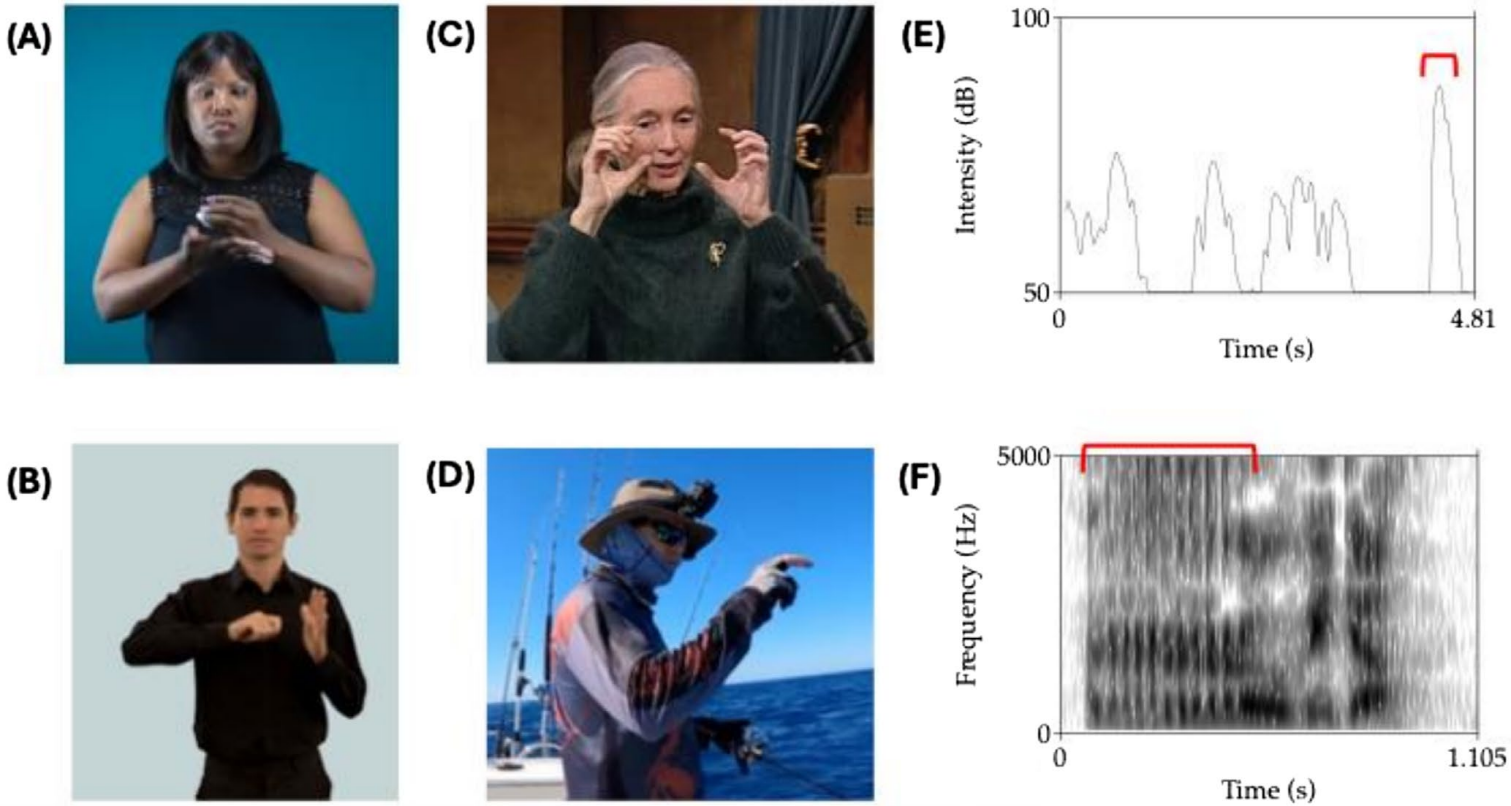
Examples of iconicity across modalities including signed language (A, B), co‐speech gesture (C, D), and speech (E, F). (A) Sign for “cup” in American Sign Language (https://aslsignbank.com/dictionary/gloss/388.html). The C‐handshape reflects the action of holding a cup. Its aperture can be modified to express “mug,” and by tilting the hand to the lips, the action of “drink.” (B) Sign for “key” and the related actions “lock/unlock” in British Sign Language (https://bslsignbank.ucl.ac.uk/dictionary/words/unlock%20with%20a%20key‐1.html). The right hand is shaped as if holding a key, while the left hand represents the lock and door itself. (C) Co‐speech gesture from a character viewpoint (https://www.youtube.com/watch?v=_ZfCcy_CYUY, 2m57s). The speaker Jane Goodall describes her early experience viewing chimpanzees: “with my binoculars I still began to learn an awful lot about [chimpanzee behavior].” The gesture occurs with “binoculars.” (D) Co‐speech gesture from an observer viewpoint (https://www.youtube.com/watch?v=XYH9YDRSYjM, 32m03s). A fisherman describes a bite: “it felt like a snappy bite though: Doonk doonk doonk.” The hand represents the striking fish, tapping forward with each “doonk.” (E) Plot of a speaker's voice intensity as he describes a play from a basketball game (https://www.youtube.com/watch?v=G4iAHlP21q0, 1m01s). The *y*‐axis shows intensity in decibels, and the *x*‐axis shows time in seconds. The speaker says, “It was one of the loudest sounds I ever heard: Boom.” The red bracket corresponds to the heightened intensity of the word “boom.” Across spoken languages, back vowels as the vowel in “boom” tend to express large magnitude compared with high front vowels (as in “teeny”) that express small magnitude (Blasi et al. 2016; Winter and Perlman 2021). (F) Spectrogram of the Italian word *rubido*, glossed as “rough” in English. The bracket shows the energy fluctuations that correspond with the initial alveolar trill. Across spoken languages, words for “rough” are disproportionately likely to contain this speech sound, which is iconic of rough texture (Winter et al. 2022).

For much of the history of linguistics, language has been regarded as a symbolic code, characterized by the doctrine of the “arbitrariness of the sign” (de Saussure 1983). In this paradigm, researchers searching for linguistic precursors in the communication of other animals have tended to search for evidence of syntax (e.g., Allen et al. 2019; Arnold and Zuberbühler 2006; Terrace et al. 1979) and the ability to use referential symbols (e.g., Pilley and Reid 2011; Seyfarth et al. 1980; Suzuki and Sugita 2024). However, in linguistics and cognitive science, there is growing support for the theory that language is fundamentally multimodal (Perniss 2018) and characterized by semiotic diversity (Kendon 2014). This research highlights the importance of *iconicity*—when the form of a signal (e.g., a word, sign, or gesture) is perceived to resemble an aspect of its meaning, whether from the perspective of its production or its interpretation (Winter et al. 2026; see Figure 1). Considerable evidence now indicates that iconicity plays vital roles in the processing, learning, historical development, and evolution of language, spoken and signed.

This iconic turn in our understanding of language raises a critical question for those interested in its origins: do our closest living relatives, the great apes, use iconic signals? The answer to this question turns out not to be straightforward. All species of great apes (henceforth “apes”)—including chimpanzees, bonobos, gorillas, and orangutans—use large, flexible repertoires of gestures (Byrne et al. 2017; Tomasello and Call 2019), and researchers have long observed that many of their gestures *appear* to be iconic (e.g., Köhler 1925; Yerkes 1943). But according to the currently authoritative theories of ape gesturing, this iconicity is illusory—an artifact of the ritualization of effective actions, either through phylogeny or ontogeny. However, to date this claim has remained mainly in the realm of speculation, with limited attempt to carefully examine ape gestures for iconicity. This paper is the first to provide a comprehensive review of the evidence regarding iconicity in ape gestures in comparison to alternative accounts of ape gesturing. Reasoning from cognitive theories of human gesture and iconicity as rooted in sensorimotor simulation and mental imagery, it concludes that apes do, in fact, show evidence of iconicity in their gestures. Thus, we find the rudiments of language in the iconic gesturing of our closest relatives, revealing a fundamental—but to date, under‐appreciated—continuity between humans and apes in the evolution of language.

## 2 Language Is Multimodal and Iconic

Language, long thought limited to speech—articulated specifically by the vocal apparatus and perceived as an acoustic signal (e.g., Hockett 1959)—is now widely understood to be a fundamentally multimodal system (Cohn and Schilperoord 2024; Enfield 2009; Hagoort and Özyürek 2025; Perniss 2018; Pleyer et al. 2025). Language is multimodal in two ways. First, it is modality‐flexible, readily implemented in speech and sign (i.e., as visible gesture) (Goldin‐Meadow 2003), and recent studies suggest even that tactile languages are possible among communities of deafblind individuals (Edwards and Brentari 2020). Second, even “spoken” language is multimodal because it is not just vocal, but expressed through the deeply integrated coordination of vocalization with manual gesture, facial expression, and other bodily movement, and it is perceived both auditorily and visually (Vigliocco et al. 2014). With the realization that language is multimodal has come mounting evidence of its widespread and pervasive iconicity (Clark 2016; Hodge and Ferrara 2022; Kendon 2014).

For much of the history of linguistics and cognitive science, researchers have focused on the *arbitrary* nature of linguistic signs, treating spoken words as pure “symbols” that rely entirely on a conventional association between form and meaning (cf. Peirce 1974). However, researchers increasingly recognize that iconicity is also a fundamental part of the design of language and how it is used (Dingemanse et al. 2015; Perniss et al. 2010). Figure 1 shows examples of iconicity in sign language (Figure 1A,B), speech (Figure 1E,F), and co‐speech gesture (Figure 1C,D). In all languages, spoken and signed, iconicity operates across levels of linguistic structure, spanning syntax (Haiman 1985), lexicon and morphology (Winter et al. 2024), phonology (Sidhu 2025), phonetics and prosody (Perlman 2026). Iconicity is also an essential property of the representative gestures that are now understood to form a deeply integrated system with spoken and signed languages (Kendon 2004; Liddell 2008; McNeill 1992), including depictive devices like quotation (Clark and Gerrig 1990) and constructed action (Cormier et al. 2015). Growing experimental evidence shows how iconicity plays vital roles in language processing (Sidhu et al. 2019; Thompson et al. 2009), language learning (Kantartzis et al. 2011; Lockwood et al. 2016; Ortega 2017), the historical development of languages (Blasi et al. 2016; Padden et al. 2013; Winter et al. 2022), and in the formation of symbol systems (Erben Johansson et al. 2021; Fay et al. 2013; Macuch Silva et al. 2020; Perlman et al. 2015).

### 2.1 Iconic Gestures

With respect to language evolution, iconic *gestures* are widely recognized as the most likely modality for an iconic precursor shared between humans and apes. In this context, *iconic gestures* refer to the spontaneous expressive movements that people (and possibly apes) produce with their hands and other bodily effectors when they communicate with each other, specifically those movements that somehow resemble, depict, or otherwise directly represent their meaning through their form. Broadly construed, iconic gestures may occur with speech, or they may function more independently, as for example, pantomime.

The “linguistic” status of the iconic gestures people use when they speak is debated, mainly on theoretical and/or philosophical grounds (Kendon 2014). Whatever the conclusion, there is ample empirical evidence that they are a universal and critical component of human communication, and one that is deeply connected to our capacity for language. People from all cultural and linguistic backgrounds commonly use iconic gestures in coordination with speaking and signing (Kendon 2004). Even people blind from birth spontaneously produce iconic gestures—for example, tilting a “C‐shaped hand in the air as though pouring liquid from a glass”—without ever having seen others produce them (Iverson and Goldin‐Meadow 1998, 228). In the case of signed languages, we know from observation that iconic gestures play a fundamental role in their creation (Senghas et al. 2004), providing the raw material that is conventionalized—lexicalized and grammaticalized—into a linguistic system. These observations of the universality of iconic gesture and their role as the source of signed languages contribute key evidence to gesture‐first accounts of language origins (Arbib et al. 2008; Armstrong and Wilcox 2007; Corballis 2003; Goldin‐Meadow 2016; Tomasello 2008; Zlatev et al. 2020).

From a psychological perspective, what makes a gesture iconic is its direct connection to the sensory and motor imagery that is activated as people produce and understand meaningful utterances (Hostetter and Alibali 2018; Kita 2000; McNeill 1992; Perlman and Gibbs 2013b). Iconicity in this sense refers to the active influence of this imagery in shaping the gestural form that is produced, or conversely, the active influence of the form in shaping how it is understood (Winter et al. 2026). While iconic gestures are subject to a degree of convention, they are characteristically dynamic and spontaneous: their form varies according to the details of the gesturer's particular meaning, and in turn, these variations contribute to differences in how the communication is understood (Cartmill et al. 2012). For example, Cook and Tanenhaus (2009) asked participants to solve the Tower of Hanoi problem, either in the real world or using a mouse on a computer, each involving a different movement trajectory of their hands. When participants subsequently described their solutions, they spontaneously produced iconic gestures that reflected the particular kinematics involved in solving their version of the problem, even as this difference was not mentioned in their speech. Listeners, in turn, were sensitive to this tacit information conveyed through iconic gesture: when they performed a computer version of the task after observing an explanation of the real‐world task, they moved the mouse with more real‐world‐like trajectories.

Iconic gestures are fundamentally creative in nature, enabling people to transmute their thoughts into spontaneously formed, expressive movements that are understandable to others. They can be used to express a wide and open‐ended range of meanings, from pantomiming actions to depicting the shapes of objects to representing more abstract ideas through metaphor (Cienki and Müller 2008). People often conceive the iconic gestures they produce from different perceptual perspectives, such as from the viewpoint of a character performing an action or of an observer viewing it (Cartmill et al. 2012). Such different perspectives impose different demands on the gesturer's imagination, including different kinds of mappings between the gesturer's body and the aspect of meaning that is being represented. In character viewpoint gestures, for instance, the gesturer's hands and body may represent the hands and body of the character, mimicking the specific kinematics of a particular context and physical situation. In observer viewpoint gestures, the hands can be molded to represent the shapes of different kinds of things, for example, an instrument that is part of the action (e.g., index and middle fingers extended in an opening and closing movement for “scissors”).

In studies of iconic gesture—and of iconicity and language more broadly—it is important to distinguish iconicity as a psychological phenomenon from its fossilized imprint on conventional forms, including signs and words, as well as gestural emblems. Consider, for example, the commonly used index‐thumb pinching gesture used to convey “small.” Such a gesture appears iconic, and certainly has iconic origins, but it is also highly conventionalized (even existing as an emoji). In any given instance of the gesture, the gesturer may be expressing a heightened, detailed sense of smallness, modifying the form of the gesture in a particular way to express its particular quality. Alternatively, the gesture may be produced mainly based on the conventional association between its form and a generic notion of small size. Similarly, in understanding the gesture, an observer might interpret it as a specific depiction of a particular instantiation of smallness (not necessarily intended by the gesturer), or they may understand the gesture primarily as a categorical convention referring generically to small size. Such dynamic interplay between iconicity and convention occurs not just in gestures, but across modalities, including iconic vocalizations and spoken words (e.g., “wild” vs. “tame” onomatopoeia; Rhodes 1995).

Despite the sense that iconicity is intuitive, and despite its critical role in bridging communication between people who lack a shared language, the use of iconic gestures does not seem to be “easier” than the use of symbols (Cartmill 2025). To the contrary, young children begin to use words before they show clear ability to produce and understand iconic gestures—at least, in the psychologically active sense. Sensitivity and appreciation of iconicity in gesture is not clearly documented until around the age of 2 years (Bates 1979; Özçalışkan and Goldin‐Meadow 2011; but see Green et al. 2023). And while studies of apes show that, with proper training and/or experience, they can master many key components of symbolic communication, including specifically the use of “arbitrary” symbols (Patterson 1978; S. Savage‐Rumbaugh et al. 1986; Terrace et al. 1979), the extent to which they can use iconic communication is disputed. In this light, the question of whether our closest living relatives share our capacity for iconic gesturing is critical to understanding the evolution of language, perhaps even more so than research into traditionally recognized linguistic properties like arbitrariness (e.g., Watson et al. 2022).

## 3 The Iconic Gestures of Apes

### 3.1 Ape Gestures

Many researchers of ape communication have pointed to their gesturing as a strong candidate for a precursor to human language, particularly in comparison to their vocalizations. Apes use a large repertoire of gestures to communicate about various functions, from initiating grooming, sex, and play, to coordinating travel and joint locomotion, to requesting food (Byrne et al. 2017; Tomasello and Call 2019). They commonly employ these gestures intentionally and flexibly, and in socially sophisticated ways. For example, ape gestures exhibit “disassociation between means and ends”—different gestures may be used for the same function, and similar gestures may be used for different functions (Call and Tomasello 2007). Gesturing apes are also sensitive to their audience's state of attention, more likely to produce visible, rather than auditory (e.g., clapping, stomping) or tactile (e.g., pulling, pushing) gestures, when their audience can see them (Hobaiter and Byrne 2011; Poss et al. 2006). And apes have the ability to modify their gestures and learn new ones, especially in captivity (Gardner and Gardner 1969; Miles 1990; Patterson 1978; S. Savage‐Rumbaugh et al. 1986), but also in the wild (Kalan et al. 2025). In these respects, it has been widely claimed that apes' gesturing is much more like language than their vocal behavior, which in comparison, has often been described as involuntary and reflexive, and not subject to modification or learning (e.g., Tomasello and Call 2019; although accumulating evidence indicates this is overstated, Perlman 2017). Thus, given the importance of iconicity in human communication, the question of whether apes can create and use gestures that are specifically homologous to human *iconic* gestures is critical to understanding the evolutionary advantages of gesture in the early origins of language.

### 3.2 Early Observations of Iconicity

While there has been little direct study of iconicity in ape gestures, there is a long history of anecdotal observations of apes producing iconic gestures (Crawford 1937; K. J. Hayes and Hayes 1951; Köhler 1925; Ladygina‐Kohts 2002; Yerkes and Yerkes 1929). Beginning with the earliest studies of apes in captivity, researchers have described their use of what appeared to them clearly to be iconic gestures. For example, Köhler described the “mimetic” gestures of chimpanzees (Köhler 1925, 307–308):

[A] considerable proportion of all desires is naturally expressed by slight initiation of the actions which are desired. Thus, one chimpanzee who wishes to be accompanied by another, gives the latter a nudge, or pulls his hand, looking at him and making the movements of “walking” in the direction desired. One who wishes to receive bananas from another initiates the movement of snatching or grasping, accompanied by intensely pleading glances and pouts. In all cases their mimetic actions are characteristic enough to be distinctly understood by their comrades.

Similarly, Yerkes and Yerkes noted that it would “indeed be difficult to exaggerate the practical importance of … pantomime … in the daily life of the chimpanzee” (Yerkes and Yerkes 1929, 308–309).

The earliest research projects with enculturated apes also described their production of iconic gestures, including human‐like pantomimes (K. J. Hayes and Hayes 1951; Ladygina‐Kohts 2002). For example, Hayes and Nissen (1971, 107) described the iconic gestures of the young chimpanzee Viki, whom they attempted, unsuccessfully, to teach to speak:

Watching bread being kneaded, she begged for a sample of dough by going through the kneading motions for a while, and then holding out her hand, palm up, moving her fingers in the gesture which means “give me” to both her species and ours. A similar incident occurred during the weekly ironing as she grew impatient for her turn to do the napkins. She stood on a nearby table, moving one clenched fist slowly back and forth above the ironing board while her other hand tried to take the iron away from “mamma.”

These anecdotal descriptions provide qualitative detail to the researchers' impressions that the apes commonly used iconic gestures. In the nearly 100 years since, researchers have continued to note the use of iconic gestures by apes, most frequently in captivity (e.g., Tanner and Byrne 1996), and especially in human‐enculturated circumstances (e.g., S. Savage‐Rumbaugh et al. 1986), but also in more free‐ranging conditions (e.g., Pika and Mitani 2006). Most often, these gestures are imperative in function, directing the behavior of another by representing the movements and actions they would like them to perform. These iconic gestures can be classified as one of three types according to the modality of communication (e.g., tactile vs. visual) and the type of iconicity they exhibit, which may reflect an increasing degree of conceptual complexity and symbolic abstraction (Perlman et al. 2014).

### 3.3 Kinds of Iconic Gestures

**FIGURE 2:**
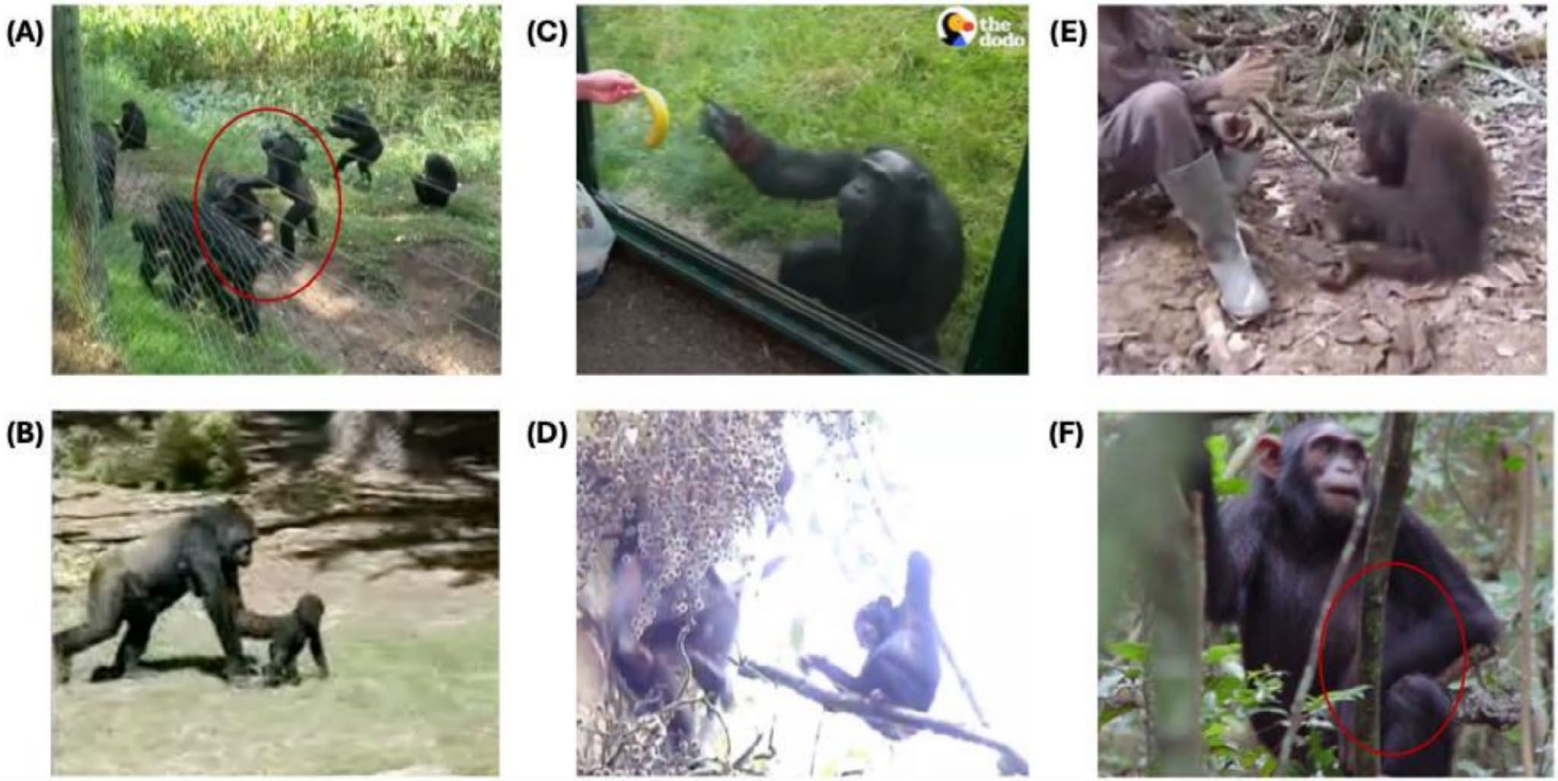
Images of apes producing different kinds of gestures that may be iconic. (A) Directive touch by a bonobo at the Lola Ya Bonobo sanctuary, image adapted from video published with supplemental materials of Genty and Zuberbühler (2015). The male touches a female on the outside of her shoulder and pushes her upper torso away to induce her to turn around and assume an orientation for dorso‐ventral copulation. The red circle indicates the two bonobos of interest. (B) Zoo‐housed gorilla produces a directive touch to guide the movement of her infant. Gesture described in Perlman et al. (2012). (C) Visual directive produced by a zoo‐housed chimpanzee, image adapted from https://www.youtube.com/watch?v=SG8d52cVG_E. The chimpanzee points at the banana and then swings its hand and arm upward, apparently to encourage the zoo‐goer to toss the banana over the barrier into the enclosure. (D) Visual directive produced by a wild chimpanzee (Great Ape Dictionary; see Graham and Hobaiter 2023). The young male produces a beckoning gesture towards a female. Image adapted from https://www.youtube.com/watch?v=NEDP12g_FRE. (E) Action pantomime by a free‐ranging orangutan at a rehabilitation site, described in Russon and Andrews (2011). The orangutan feigns chopping at a coconut with a palm frond to request a human caregiver to crack it open with a machete. Image adapted from video in https://www.bbc.co.uk/news/science‐environment‐10926301. (F) Possible instance of pretend play (Kahlenberg and Wrangham 2010). A 9‐year‐old female chimpanzee carrying a stick, possibly pretending as if it is an infant. The red circle indicates the stick in the chimpanzee's left “groin pocket.” Image adapted from fig. S1 of Kahlenberg and Wrangham (2010). *Photo:* Sonya Kahlenberg.

The most frequently observed iconic gestures are *directive touches*. In various close‐range circumstances—such as in negotiating play, sex, joint travel, or grooming—an ape may want another ape to move in a particular way, either into a particular position or orientation, or to a particular location (see Figure 2A,B). To achieve this, the initiating ape may use directive touches to guide the recipient's movement and positioning, such as by giving them a light push, pull, nudge, tug, or lift. The location of the touch along with its direction and implied force communicates the direction and type of movement that is desired.

For example, Perlman et al. (2012) described the directive touches used by a captive mother gorilla—specifically pushes of different orientations (backhand, inside out, overhand, underhand) along with other tactile gestures and actions (grabs, pulls, swings, carries) to guide her infant around their enclosure. These gestures varied in form in ways that were tuned to the physical interaction, appearing to resemble a partially enacted version of the physically effective pushes that would serve to move the infant in the desired way. Similar gestures, described as “positioning motions,” were used by captive bonobos as they negotiated sexual positions (E. S. Savage‐Rumbaugh et al. 1977), exemplified in a sequence of photos depicting an interaction between a young male bonobo and an older female in which the male attempts a series of gestures to orient the female for copulation (Savage‐Rumbaugh et al. 1977, 103–107). For instance, in one gesture, the male gently “touches the female's shoulder, pushing her upper torso away from himself to induce her to turn around.” Savage‐Rumbaugh and colleagues described generally how “the body orientation of the initiator and the recipient determined the exact topography of the gesture in each instance” (E. S. Savage‐Rumbaugh et al. 1977, 108).

*Visual directives* make up the second kind of iconic gestures produced by apes. These include various beckoning, reaching, and begging gestures, which appear to be motions made in the air that are similar to tactile directional and positioning gestures, but guide how the recipient should move or what act they should perform without directly touching them (see Figure 2C,D). These kinds of gestures have been described as exhibiting a degree of symbolic abstraction beyond tactile directive gestures (E. S. Savage‐Rumbaugh et al. 1977). They occur in similar contexts to directive touches—negotiating grooming, sex, play, or travel—but may be used when the interacting apes are beyond arm's reach of each other.

For example, Tanner and Byrne (1996) observed the visual directive gestures performed by a captive silverback gorilla to coordinate playful action with a younger female. His gestures appeared to illustrate the future actions that he desired of her by “drawing” the relevant path of motion in space or on her body. For example, to invite sexual contact and play, he tended to complete a three‐part phrase in which he would tap her, perform an *armswing under* gesture, followed by touching his genital area (usually accompanied by playface). In the sexual solicitations of wild bonobos, Genty and Zuberbühler (2014) observed the use of beckoning gestures, described as “stretching the arm toward a recipient followed by a sideways sweeping movement of the arm toward the self and ending with a twirl of the wrist from palm upward to downward.” They were typically preceded by a sexual initiation posture and followed by a pivot in the direction of desired location for sexual contact. Similar gestures, called “iconic hand motions” were used by captive bonobos in their negotiation of sexual positions (E. S. Savage‐Rumbaugh et al. 1977).

Other examples of visual directives include palm‐up begging gestures for desirable food that resemble the act of reaching for food or taking it from the mouth of a peer, possibly even gently touching the recipient's mouth (Bard et al. 2019; Goodall 1986). In captivity, apes often produce whole‐handed “pointing” gestures to human caregivers, typically to direct their attention towards some desired object (e.g., food) that is on the other side of a mesh fence (Leavens 2021). The gesturer extends their arm out towards the target, possibly putting their fingers through the mesh. These gestures function as imperative pointing gestures, and they are typically analyzed as indexical rather than iconic gestures. However, in the present context, these may be seen alternatively as iconic reaches towards the object, even as their intention is to direct the recipient's attention.

In some instances, researchers taking note of the iconicity of visual directives have interpreted the gesturer's hand as a representation of the recipient's body, forming a temporary equivalence between the motion of the hand and the movement of the recipient. For example, Savage‐Rumbaugh characterized “iconic hand motions” used by bonobos by stipulating, “the hand is not acting as a hand in the instance of gesturing, but as a symbol for the recipient's body.” However, in many if not most instances, these are more plausibly representations of the directive action from the perspective of the actor, enacting the manipulative action that could coerce the recipient to move in the desired way.

Finally, the third kind of iconic gesture that apes produce—and arguably the most semiotically sophisticated—is *pantomimed actions*. These are gestures like the previously described bread‐kneading and ironing gestures used by the human‐enculturated chimpanzee Viki. In visual directives, the gesturer creates an iconic representation of an effective action they would perform directly on the recipient to solicit their response, as if to the force of the actual action. In pantomimed actions, the iconic gesture represents an action with the communicative intention either to request from the partner an object associated with the action, or to solicit the partner to themselves perform the action (see Figure 2E).

**FIGURE 3:**
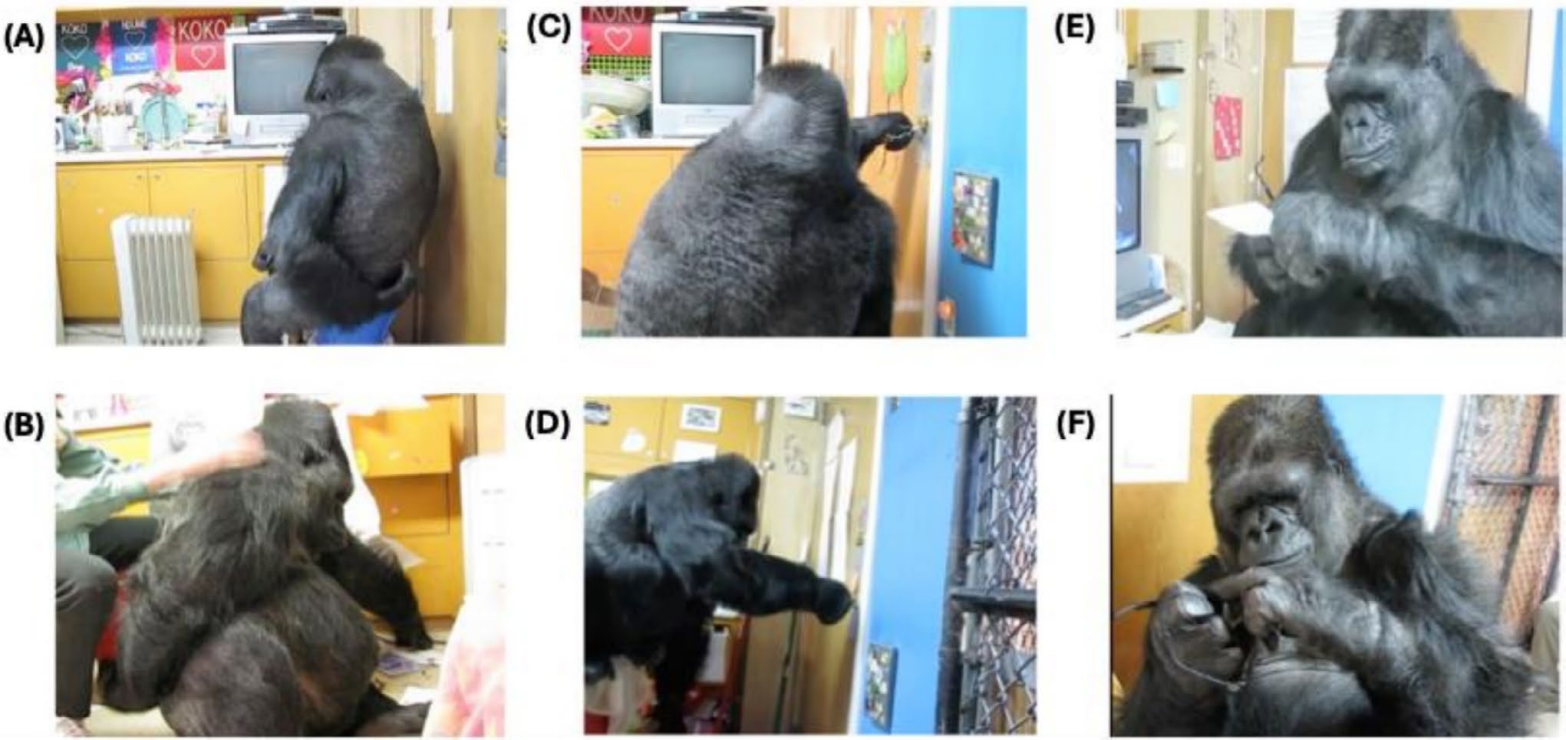
The enculturated gorilla Koko (37–39 years of age) performs different instrumental actions (top row) and corresponding pantomimes (bottom row), described in Perlman and Gibbs (2013b). (A) Koko scratches her lower back, using vigorous, audible scratches. (B) Koko produces a gentler communicative scratch after a glance back at her caregiver, requesting her caregiver to scratch her back instead of the current massaging action. (C) Koko makes an instrumental attempt to unlock the door with a key. The action is marked by persistence and extended focused attention on the lock. (D) Koko pantomimes the act of unlocking a door gesture with a key, perfunctorily touching a key to the lock to request her caregiver to perform the action, leading to a walk outside. (E) Koko wipes the lenses of eyeglasses with a tissue (after huffing on them), a routine she learned from human caregivers. (F) Koko, lacking a tissue, performs a perfunctory wipe with her finger, apparently to request from her caregiver a tissue to properly complete the act.

**FIGURE 4:**
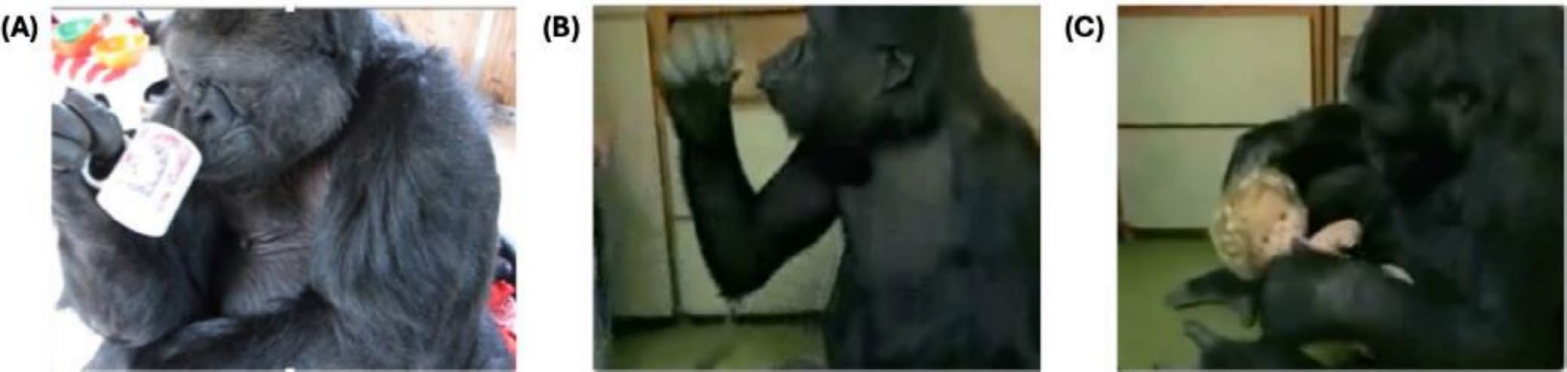
Koko performs similar drinking pantomimes in contexts ranging from communication to pretend play with a doll. (A) Koko (~38 years of age) performs a drinking action with an empty mug (Perlman and Gibbs 2013a). She brings the mug to her lips and tilts it back with slurping sounds, apparently to communicate to her caregiver that she would like the mug to be filled with a drink. (B) and (C) show images of Koko (~4 years of age) from the documentary film *Koko: A Talking Gorilla* (Schroeder, 1978). (B) Koko pretends to drink from the small plastic cup of a toy doll, producing similar slurping sounds to those produced in (A). (C) Koko playfully inserts the cup in the doll's mouth, shortly before extending the cup to her caregiver to invite her to also “take a drink.”

Pantomimed actions are used most variably by enculturated apes, typically to communicate with humans about action routines learned from their shared history of interaction and cultural experience. The apes often perform these gestures with the objects and people relevant to the routine in view, and the gestures may be directed at them. For example, Savage‐Rumbaugh and colleagues described the gestures produced by the enculturated bonobos Kanzi and Mulika, who “made twisting motions toward containers when they needed help in opening twist‐top lids” and “hitting motions toward nuts they wanted others to crack for them” (S. Savage‐Rumbaugh et al. 1986, 218). In some cases, the apes perform partial or ineffectual renditions of the routine that include physically manipulating the relevant objects. For example, Figures 3A–F and 4A–C show some of the pantomimes produced by the gorilla Koko when she was nearly 40 years of age, with a life history of intensive human enculturation since infancy (Perlman and Gibbs 2013a). Outside of human influence, the use of pantomimed scratches, directed on the gesture's own body to request a partner to groom that particular area, has been observed on multiple occasions by free ranging chimpanzees (Goodall 1986; Pika and Mitani 2009), as well as by enculturated apes as Koko in Figure 3a (Perlman and Gibbs 2013a). A study of wild female bonobos observed their use of a *hip shimmy*, resembling an action involved in genito‐genital rubbing (Douglas and Moscovice 2015). Orangutans have also been observed to use action pantomimes. A study of free‐ranging rehabilitant orangutans identified 18 clearly defined pantomimes from 20 years of written field observations (Russon and Andrews 2011). Most of the pantomimes—which were mainly performed with human partners, rather than other orangutans—related to requested actions or objects, shared events, and actors' abilities or intentions. For instance, an orangutan feigned an inability to open a coconut, chopping at it ineffectually with a palm petiole as a request for a human to cut it with a machete.

Researchers have noted the close kindship between the use of pantomimes for communication and the production of similar behaviors during pretend play (Green et al. 2023; Russon 2016), which are both rooted in a similar acting “as if” (Kendon 1991) (see examples in Figure 4A–C). As with communicative pantomime, the research literature contains various descriptions of apes engaging in pretense during play, especially those with human enculturation. For example, Hayes (1951) described the chimpanzee Viki at 18 months of age playfully tugging an imaginary pull‐toy around by its imaginary rope, even pretending it had gotten caught on a piece of furniture and soliciting her human mother's help to free it. A more formal study described the development of representational play in enculturated chimpanzees and bonobos, finding multiple video‐recorded instances of full‐blown pretense, including, for example, imaginative play grooming with a monkey puppet or pretending to eat fruit displayed in a picture (Lyn et al. 2006). There is some evidence of apes engaging in pretense in the wild, too, although evidence is more limited. One prominent example is observations of juvenile chimpanzees, especially females, carrying sticks “in a manner suggestive of rudimentary doll play,” perhaps as pretend play at mothering (Kahlenberg and Wrangham 2010; see Figure 2F). Chimpanzees have also been observed using demonstrative actions for teaching: for example, Boesch et al. (2019) described a mother demonstrating to her daughter the use of an anvil to crack open nuts.

Thus, from nudges to beckons to pantomimes, many researchers over the years have taken the iconicity of ape gestures to be self‐evident. In the current day, researchers generally agree that many of the gestures apes produce do, at least, *appear* iconic—that is, they clearly resemble effective actions associated with their goal or meaning. Of dispute, however, is the psychological relationship between these gestures and the actions they resemble, and the nature of the process that leads to this resemblance (Perlman et al. 2012). Are these ape gestures truly iconic in a homologous sense to the spontaneous iconic gestures of humans?

### 3.4 Doubts About Iconicity

In recent decades, the authoritative answer to the question of whether apes use iconic gestures has been negative—that any apparent iconicity is illusory, evident in the eye of the researcher, but not in the eyes of the gesturing apes (Arbib et al. 2008; Byrne et al. 2017; Tomasello and Call 2019). Originally, this argument was put forward on theoretical grounds that apes lack the cognitive capacities—both for analogical reasoning and for reasoning about other minds and sharing joint attention with them—that are needed to produce and understand an iconic gesture. Tomasello (2008) considered that, “To use an iconic gesture one must first be able to enact actions in simulated form, outside their normal instrumental context—which would seem to require skills of imitation, if not pretense. But even more importantly, to comprehend an iconic action as a communicative gesture, one must first understand to some degree the Gricean communicative intention; otherwise the recipient will suppose that the communicator is simply acting bizarrely, trying to run like an antelope or to dig a hole for real when the context is clearly not appropriate” (p. 233). Experiments have shown that, without extensive practice with humans (Custance et al. 1995; Hayes and Hayes 1951), apes find it difficult to imitate precise bodily movements of others, tending to focus more on the outcome than specific details of the action itself (Buttelmann et al. 2013). Experiments have also shown that while apes gesture intentionally to manipulate the behavior of others, they tend not to gesture cooperatively and establish shared intentionality with their partner. More recently, the argument against iconicity has found support in experimental studies directly testing the ability of apes to understand and produce iconic gestures (Bohn et al. 2016, 2019, 2020; Dezecache et al. 2019; Grosse et al. 2015). Under experimental conditions, chimpanzees and bonobos showed scarce evidence they could produce iconic gestures to inform a human cooperator how to retrieve food from a mechanical apparatus (Grosse et al. 2015). In comparison, 2½‐year‐old children successfully used iconic gestures in a comparable task. In another experiment, apes also failed to understand and make use of iconic gestures produced by humans (Bohn et al. 2020). Two‐year‐old and especially 3‐year‐old children showed some ability to use iconic gestures produced by adults to learn to perform a novel action to open an apparatus, whereas apes failed to take advantage of the gestured information.

Given these cognitive limitations—at least, as revealed under experimental conditions—Tomasello, Call, and colleagues argued that despite how ape gestures may appear to human eyes, they cannot plausibly be iconic for the apes themselves. Instead, they proposed that many ape gestures appear iconic because they “derive from attempts to actually move the body of the other in the desired direction” (Tomasello and Call 2019, 467)—a process they called ontogenetic ritualization.

## 4 Iconicity Versus Ritualization

### 4.1 Ontogenetic Ritualization

Plooij (1978) first described how the gestures of chimpanzee infants may form “as rituals developed over the many, regularly recurring, social practices between mother and baby” (p. 130). For example, Plooij observed that, in the earliest interactions between a young infant and their mother, the mother might lift the infant's arm to groom their side and armpits. By the age of 6½ months, the infant may participate in grooming by raising their arms voluntarily to make space for their mother to groom. And at 11 months, an infant was seen to initiate the grooming session by the *arm‐raise* gesture. Building on Plooij's observations, Tomasello and colleagues proposed that this essential process of ontogenetic ritualization is the primary means by which apes develop their large and flexible gestural repertoires. Originally non‐communicative, instrumental behaviors—carried out as part of dyadic activities like play, grooming, joint travel, and sex—are shaped over repeated interactions into ritualized gestures that resemble abbreviated portions of the original behavior. Ontogenetic ritualization can be formalized as involving the following sequence of steps (Gasser et al. 2014):

- Individual A performs behavior X (not a communicative signal), and individual B consistently reacts by doing Y.
- B anticipates A's overall performance of X by starting to perform Y before A completes X.
- Eventually, A anticipates B's anticipation and produces an initial portion of X in a ritualized form X^R^ to elicit Y.

The result is a gesture X^R^ that resembles, to some extent, the behavior X from which it is derived. Thus, Tomasello and colleagues proposed that what appear to be iconic gestures produced by apes are most likely “garden‐variety ritualized behaviors” (e.g., Tomasello and Call 2019, 467).

However, evidence for ontogenetic ritualization is indirect, largely due to the difficulty of conducting the kind of continuous, longitudinal observations required to study such a process. Consequently, the specific temporal dynamics of ontogenetic ritualization—such as the timespan and number of interactions required for an action to become ritualized into a gesture—have remained unspecified, speculated only to occur over “considerable” time (Halina et al. 2013). Addressing this issue by seeking more granular evidence for ontogenetic ritualization, Halina and colleagues focused on the gestures used by mother and infant bonobos to initiate carries for joint travel. Their study examined 10 mother–infant dyads across six captive sites, with observations beginning when the infants were about 10 months of age and spanning 6–10 months per dyad, with hundreds of hours of total observations. The authors hypothesized that by ontogenetic ritualization, gestures should resemble the particular actions from which they were derived. In particular, the gestures should reflect the differential roles that mothers and infants play in the carry interaction, and further, that variability between individual gestural repertoires should reflect variability in carrying interactions between the mother‐infant dyads. The findings showed that many of the gestures used—including, for example, various grabs, touches, raisings of limbs, and presentations of backs and venters—plausibly reflected elements of actions involved in initiating carries. Some of these gestures, such as *spin body* and *step foot*, were specific to infants, and others such as *present back* and *present venter* were specific to mothers—appearing to reflect their respective roles. Interestingly, only infants from one of the groups observed used a *spread legs* gesture, which resembled the act of an infant wrapping their free legs around their mother for a carry as when hanging down from above. It was noted that the infants in this group, compared with the others, most frequently entered into carries from a hanging position. Overall, Halina and colleagues interpreted these findings as evidence that the gestures were derived from ontogenetic ritualization.

Notably, however, the finding that gestures resemble the specific actions to which they are related is also entirely consistent with the hypothesis that they are iconic. In Halina and colleagues' study, this possibility was dismissed a priori on the assumption that apes are not capable of using iconic gestures. Instead, the study sought to distinguish their results from the other dominant hypothesis proposed to explain the large, flexible gesture repertoires of apes—that ape gestures are biologically inherited, derived from the evolutionary process of *phylogenetic* ritualization.

### 4.2 Phylogenetic Ritualization

The ontogenetic ritualization hypothesis predicts idiosyncrasy in ape gestural repertoires, with variability arising between dyads that ritualize gestures through their individualized interactive routines. And in the relatively small‐scale studies carried out by Tomasello, Call, and colleagues, mostly on captive groups of apes, there appeared to be a substantial amount of idiosyncrasy across individual repertoires. However, this evidence for ontogenetic ritualization has been challenged by a series of wider studies conducted by Byrne, Hobaiter and colleagues, which included long‐term field studies in addition to studies of captive groups (Byrne et al. 2017). In contrast to Tomasello, Call and colleagues, these studies found strikingly little variability across gestural repertoires. With enough observation time, gestures that at first appeared idiosyncratic were eventually found to be used by others too, suggesting that previous variability in repertoires was due to under‐sampling.

Indeed, the studies of Byrne, Hobaiter and colleagues documented extensive gestural repertoires typical of each species of ape: 66 gesture types in chimpanzees (Hobaiter and Byrne 2011), 68 in bonobos (Graham et al. 2017), 102 in gorillas (Genty et al. 2009), and 64 in orangutans (Cartmill and Byrne 2010). Gesture types were distinguished in fine‐grained granularity according to whether they elicited similar responses from the recipient (Byrne et al. 2017), which included “meanings” like grooming initiation, acquiring an object, following me, sexual attention, stopping that, repositioning the body, and attending to a specific location. The repertoires include 36 gesture types found common to all species of great apes, suggesting they may be shared by common descent. Among these ape‐universal gestures were, for example: *arm(s) raised*, *arm(s) waved*, *beckoning*, *reaching palm*, *reaching wrist*, *hand(s) flung*, *poking*, *grabbing*, *grabbing‐pulling*, *pushing*, *pushing directed*, *tap other*, and *touching other*. Despite the large size of the different species' repertoires, Hobaiter and Byrne (2017) determined they represent just a small fraction (~12%) of an estimated 1000+ anatomically possible gestures that apes could physically produce. Based on their findings across ape species, Byrne, Hobaiter and colleagues argued the most parsimonious explanation is that ape gestural repertoires are biologically inherited, winnowed through natural selection into species‐typical forms and messages (Byrne et al. 2017). On this hypothesis, the gestures would have evolved by phylogenetic ritualization, the same process as the signal repertoires of other animal species (Lorenz 1966).

In ethology, animal signals and displays are generally understood to evolve from the ritualization of functional action sequences. A classic example is the snarling facial expression of many mammals (including humans; Van Hooff 2012), which is arguably derived from the practical action of retracting the lips to initiate a bite. Over generations of antagonistic encounters, natural selection favored animals quick to recognize the initial lip retraction of an antagonist as a precursor to an ensuing bite, and in turn, it favored animals who instinctively exaggerated the lip retraction as a signal they intended to bite, potentially sparing a costly physical fight. These interactions proceeded over the millennia, eventually evolving into the ritualized snarl observed across many mammals today. As a process, phylogenetic ritualization can be seen as kindred to ontogenetic ritualization: they are essentially similar processes operating on different timescales. Indeed, Tomasello, Call and colleagues originally proposed the idea of ontogenetic ritualization in direct analogy to the evolutionary process.

Two recent studies have found that humans may also share many of the biologically inherited gestures possessed by apes. One study examined the gestures of 2‐year‐old children raised in Germany and Uganda, applying a comparable procedure to that used in Byrne and colleagues' studies with apes (Kersken et al. 2019). The results showed 52 gestures within the children's repertoire, with all but two overlapping with other ape repertoires. Adult humans also appear to have access to this universal repertoire. In a video play‐back experiment, Graham and Hobaiter (2023) found participants were able to understand the meanings—at rates better than chance—of all but 1 of 10 gestures most frequently produced by chimpanzees and bonobos. The results of these studies can be interpreted as evidence that humans have retained their innate understanding of a universal repertoire of available gestures, shared across human and nonhuman ape species.

As with studies in support of ontogenetic ritualization, it is again notable that a consistent finding across these studies is the “resemblance” observed “between the communicative gesture and the physically effective action for the same result” (Byrne et al. 2017, 763). This resemblance, while consistent with both ontogenetic and phylogenetic ritualization, is also the hallmark of iconicity.

### 4.3 Accounting for Contextual Variability

To date, much of the research on ape gestures, including research in support of both ontogenetic and phylogenetic ritualization, has been rooted in the ethological tradition. In this vein, it has largely sought to classify gestures as belonging to distinct types, such as those listable in an ethogram. As a methodology, this approach abstracts away from the variability of individual gestures towards identifying their typical forms. Ritualization, whether postulated to occur through ontogeny or phylogeny, reflects this approach. As a general process, ritualization leads to signals with stereotyped forms and communicative functions (Perlman et al. 2012). Called “emancipation” in ethology, there is taken to be a categorical separation between a signal and the action from which it is derived. For example, many studies explicitly operationalize ape gestures to include only “mechanically ineffective” actions, excluding from analysis more forceful actions that may also be communicative (e.g., Hobaiter and Byrne 2011; Liebal et al. 2004).

In contrast to this approach, some recent studies have zoomed in on the finer‐grained details of how apes use gestures in context. Whereas ritualization‐focused accounts predict stereotyped gestures, these studies highlight the variability in individual ape gestures, noting how they are often tuned to the particular situation and intention at hand. For example, one study examined the use of the gesture *touch*—“an individual voluntarily using a body part to make tactile contact with another individual for a communicative purpose”—within the interactions of young chimpanzees and adults in a semi‐natural social group (Bard et al. 2019). The findings showed great flexibility and versatility in the gesture, including 36 different forms directed to 70 different locations on the body of social partners, occurring in 26 different contexts. In another study observing the variability of gestures in the development of young chimpanzees, Pika and Fröhlich (2019) concluded that many gestures—their forms and the meanings they acquire—are socially negotiated. They described an interactive process in which the creation of gestures does not begin with shortening of an action sequence, but instead through the ongoing and flexible online modification of behaviors shaped over time. The process is influenced by various contextual factors, including the anatomical and attentional affordances of the interaction (e.g., related to the distance of communication). Similarly, based on detailed qualitative analyses of ape interactions, King (2004) described ape gesturing as a dynamic dance, observing how apes continuously anticipate and adjust to each other's actions through recurring dyadic social interactions.

Moreover, while studies have often distinguished mechanically ineffective gestures from mechanically effective actions, in practice, the line between gesture and action is not so clear. For example, Perlman et al. (2012) described how the directive touches used by a zoo‐housed mother gorilla to negotiate joint travel with her infant varied in their degree of physical force. When her infant was more compliant, she used gentle gestures to communicate the direction and orientation she desired for them to move. When he was more stubborn, or when the situation was more urgent, she tended to use more physically forceful gestures with him—in the most extreme instances, even snatching him up or dragging him. Thus, gentler gestures and more forceful actions functioned together to coordinate joint movement, revealing a fluid relationship between gesture and action.

Recently, some prominent theoretical accounts have emphasized that phylogenetic and ontogenetic ritualization do not explain the variability of ape gestures—particularly, the extent of resemblance between gestures and their corresponding actions afforded in context. Scott‐Phillips and Heintz (2023) argued that apes are inclined to produce such gestures because they possess first‐order intentionality, making them able to anticipate how their behavior might elicit the desired reaction of a recipient based on expectations learned from past interactions. Thus, apes produce gestures as they seek to make a recipient aware of their intentions, such as to play, travel, have sex, or be groomed. An intuitive way for them to do this is to produce gestures based on their experience with functional actions relevant to the context, often leading to forms that bear perceptual resemblance to those actions. Similarly, Graham et al. (2025) proposed a “recruitment view” to explain the dynamic form‐function resemblance of many ape gestures. They argued that apes produce many of their gestures by recruiting features of their existing behavioral repertoire for communicative purposes: thus, gestures inherit their communicative functions from visual and tactile “presentations of familiar and easily recognizable action schemas and states and parts of the body.” However, despite their appearance, Graham and colleagues argued that such gestures are not truly iconic as the gesturing apes are not “reflectively aware” of the form‐function resemblance, and are not “guided by a metarepresentational process in which the gesture intends that her audience recognize that her gesture resembles an action that is connected to her communicative goal” (Graham et al. 2025, 196).

Thus, while highlighting the considerable contextually‐tuned variability in ape gestures, both theoretical accounts—Graham et al. (2025) and Scott‐Phillips and Heintz (2023)—nevertheless maintain a categorical distinction between the iconic‐*looking* gestures produced by apes and the truly iconic gestures produced by humans. Like previous arguments for ontogenetic and phylogenetic ritualization, these accounts hold Gricean intentionality and the capacity to understand analogy as the defining criteria of whether a gesture is iconic in the human sense. Yet, from a human cognitive perspective, these are, arguably, not the most illuminating criteria. While the iconic gesturing of humans can sometimes be a reflective process, and it is unquestionably a social activity, people often produce iconic gestures spontaneously as a natural outgrowth of how they conceive and verbalize their thoughts, often without conscious awareness and deliberation on their design. So how do the iconic‐looking gestures of apes appear from this point of comparison?

### 4.4 Ape Gestures Are Truly Iconic

Within the cognitive study of human gesture, the production of an *iconic* gesture is distinguished by the active influence of the gesturer's meaning in shaping its form, leading to a gesture that, to some degree, resembles an aspect of its meaning (Winter et al. 2026). For observers, a gesture is iconic when its form is interpreted as resembling an aspect of its meaning, guiding how the gesture is understood. From this perspective, we may see a fundamental homology between the iconic‐looking gestures of apes and the truly iconic gestures of humans.

From directive touches to visual directives to pantomimed actions, many researchers agree that apes produce gestures that are dynamically shaped according to their in‐the‐moment sense of a particular action or thing that is desired. As apes tend to produce these action‐derived gestures within their normal instrumental context, observers can understand them fairly directly as representations of those actions, “through an activated sense of the desired action within a context that is relevant to exactly that sort of action” (Perlman et al. 2012, 65). Thus, there is no need for the recipient to understand the gesturer's “Gricean communicative intention” or to establish a strict dual representation of the iconic mapping between gesture and action. The connection between form and meaning is scaffolded by context and, in many cases, rich past experiences behaving in similar situations—something that is notably lacking in laboratory experiments (e.g., Bohn et al. 2019; Grosse et al. 2015).

On this view, the iconic gestures most conceptually accessible to apes are those most “tightly connected—in form, meaning, and context—to the presently afforded instrumental and attentive actions that are available to the gesturer” (Perlman et al. 2012, 68). Psychologists studying gesture development in children have postulated a developmental cline of “symbolic distancing,” ranging from conceptually simple iconic gestures to more challenging ones (Cartmill et al. 2012; Green et al. 2023). Simplest is a “mimetic facsimile” of the referent, with decreasing “tangible likeness” between form and meaning developing over time (Werner and Kaplan 1963, 47–48). For example, one experiment found that when recounting a narrative, younger children tended to gesture more on their bodies or on the table in front of them, using the surface to represent the ground on which the narrated events took place (Sekine et al. 2018). Another experiment found that younger children were less likely to pantomime an action (e.g., tooth brushing) as if holding an imaginary instrument (e.g., a toothbrush), and more inclined to use a body part (e.g., an index finger) as a physical placeholder for it (Boyatzis and Watson 1993). A similar cline has been proposed for the emergence of iconic gestures in apes, ranking tactile directives as simplest, followed by visual directives (E. S. Savage‐Rumbaugh et al. 1977), and more complex action pantomimes (Perlman et al. 2014).

Reports suggest that apes with human enculturation may produce a wider array of pantomimed actions than apes lacking this experience. These gestures are typically related to actions and routines learned from humans, often involving human artifacts—such as the gorilla Koko's pantomimes of drinking with a mug and using a key to unlock a door. It is unclear whether these gestures of more complex actions reflect a developmental enhancement of apes' cognitive ability for iconicity when immersed in extensive human interaction. Alternatively, it could be that the ability of enculturated apes to produce iconic gestures is no more sophisticated than that of other apes; they just have a larger repertoire of actions in their lives to gesture about. Supporting this latter interpretation, it is notable that many action pantomimes of enculturated apes are produced with the relevant artifacts either in hand or perceptually accessible, suggesting their iconic capacity remains limited to cases with contextual scaffolding.

For humans, the creative power of iconic gestures is in their open‐ended ability to transmute meaning—whatever that may be—into spontaneously formed, expressive movements that are understandable to someone else (Pleyer et al. 2025). Wide‐ranging observations of apes suggest they may share with us the rudiments of this power, particularly within key domains of their social lives, such as in negotiating joint locomotion, play, grooming, and sex. Arguably, this capacity to communicate with iconic representations of actions frees apes from the limits of a ritualized repertoire, which is stereotyped and inflexible by design. Indeed, the reason apes may appear to have such large repertoires of gesture types compared with the signal repertoires of other animals is that they are not “repertoires” at all. Iconic gestures provide apes with an open‐ended means for negotiating their complex social interactions, one that is adjustable to express contextualized nuances of meaning—for example, a desire, in the moment, for a partner to assume a particular bodily orientation or to perform a particular action. On this account, the key distinction between human and ape iconic gesturing is not a categorical one—both create and understand iconic gestures by the homologous process of enacting simulated actions in context. What differs is the degree of imaginative abstraction and displacement of which they are capable, which is vast in humans compared with apes, who are heavily bounded to the here‐and‐now.

Notably, the challenge of identifying whether a gesture is “truly” iconic and the extent to which gesturers are aware of any iconicity in their gestures is not unique to the study of apes. Similar issues face human developmental research on the early language and gesturing of young children. For example, aiming to focus on intentional communication, these studies have often defined gestures in ways that categorically distinguish them from instrumental actions—for instance, excluding hand movements that act on objects even when they may be communicative (e.g., Özçalışkan and Goldin‐Meadow 2011). Like the iconic‐looking gestures of apes, iconic gestures of human infants tend to be heavily scaffolded by context, often including the handling of relevant objects (Green et al. 2023). Not surprisingly then, the use of such conservative criteria leads to older age estimates of when infants and young children begin to use and understand iconic gestures. By design, analytical procedures that categorically distinguish between iconic gestures and actions discard the rich communicative behavior that happens in between these prototypes. It may be in these transitional, context‐scaffolded margins where the origins of iconicity lie, both in ontogeny and phylogeny. By studying how the earliest iconic communication of young children develops from this rudimentary phase, future research may also gain a deeper understanding of the iconic gesturing of apes, its continuities with human communication, and the limitations that distinguish it. To this end, naturalistic and longitudinal studies, more than experiments, may be best suited for the study of iconicity within the rich interactive contexts in which it thrives.

Finally, it is important to note that the hypothesis that iconicity plays an essential role in ape gesturing is not at odds with other accounts postulating ritualization at different timescales. Almost certainly, ape gestures are molded to a degree by repetition in commonly occurring social interactions as they become routinized and abbreviated (i.e., ontogenetic ritualization). This may be comparable to human communication, which—while exhibiting iconicity—is also shaped by ritualization processes like conventionalization and grammaticalization. The role of iconicity is also completely consistent with the operation of evolutionary processes like phylogenetic ritualization, which may shape some specific ape gestures (e.g., the chest‐beating display of gorillas; Perlman and Salmi 2017). Ultimately, a complete understanding of ape gesturing will need to explain the dynamic interplay between these three different processes, each operating on different timescales.

## 5 Conclusion

Despite the long scholarly emphasis on the human ability to communicate with arbitrary symbols, it may actually be our capacity for iconic expression that stands out most in comparison to the communication of other animals. Within cognitive science and linguistics, many researchers now understand that iconicity is a fundamental property of language, both spoken and signed (Dingemanse et al. 2015; Perniss et al. 2010; Pleyer et al. 2025), and it is crucial to our ability to create new symbol systems, languages included (Armstrong and Wilcox 2007; Fay et al. 2014; Perlman et al. 2015). On this view, iconicity is not a by‐product of linguistic communication, but one of its essential components. Thus, when searching for the evolutionary precursors of language, it is crucial to ask whether apes, our closest living relatives, show any proclivity for iconicity. The evidence reviewed in this paper suggests that, in a significant way, they do. Apes commonly use a range of gestures that appear homologous to the iconic gestures of humans, even if they are comparatively restricted in imaginative scope and anchored heavily in a here‐and‐now context. As great apes, we all share a propensity to mold our limbs into meaningful representations of actions, and in turn, to make sense of these iconic representations in context. This fundamental capacity for iconic gesturing may have been a critical precursor to the evolution of language.

## Author Contributions

The author (MP) drafted and revised the manuscript.

## Funding

The author has nothing to report.

## Conflicts of Interest

The author declares no conflicts of interest.

## Data Availability

Data sharing not applicable to this article as no datasets were generated or analysed during the current study.
